# Analysis of the cost-effectiveness of surfactant treatment (Curosurf®) in respiratory distress syndrome therapy in preterm infants: early treatment compared to late treatment

**DOI:** 10.1186/1824-7288-40-40

**Published:** 2014-05-02

**Authors:** Carlo Dani, Roberto Ravasio, Leonardo Fioravanti, Maria Circelli

**Affiliations:** 1 Department of Neurosciences, Psychology, Drug Research and Child Health, Division of Neonatology, Careggi University Hospital of Florence, Florence, Italy; 2 PHarmES sas, Studi di valutazione economica, Milano, Italy; 3 Chiesi Farmaceutici, Via Palermo 26/A, 43122 Parma, Italy

**Keywords:** Surfactant, RDS, nCPAP, Cost-effectiveness, Preterm infants

## Abstract

**Background:**

The best criteria for surfactant treatment in the perinatal period are unknown and this makes it of interest to consider the possible economic implications of lessening the use of more restrictive criteria.

**Objective:**

The objective of this study is the evaluation of the costs of respiratory care for preterm infants with Respiratory Distress Syndrome (RDS) treated with "early rescue" surfactant compared to a "late rescue" strategy.

**Methods:**

The study was carried out applying the costs of materials used, of staff and pharmacological therapy calculated in the Neonatal Intensive Care Unit (NICU) of an Italian hospital to the Verder *et al*. study (Pediatrics 1999) clinical data.

**Results:**

The cost for patients treated with *early* strategy was slightly lower than for patients treated with *late* strategy (Euro 4,901.70 vs. Euro 4,960.07). The cost of treatment with surfactant was greater in the *early group* (Euro 458.49 vs. Euro 311.74), but this was compensated by the greater cost of treatment with Mechanical Ventilation (MV) in the *late* group (respectively Euro 108.85 vs. Euro 259.25).

**Conclusions:**

The cost-effectiveness analysis performed in this study shows how *early* treatment with surfactant in preterm infants with RDS, as well as being clinically more effective, is associated with a slightly lower cost.

## Introduction

The management of infant Respiratory Distress Syndrome (RDS) involves artificial respiratory support and surfactant treatment. Respiratory support includes nasal Continuous Positive Airway Pressure (NCPAP) [1] and Mechanical Ventilation (MV) [2], which are known for their effectiveness in reducing mortality and morbidity caused by RDS. However, MV is invasive and has the potential for injuring the airways and lung parenchyma. Ventilator-induced lung injury (VILI) may be associated with alveolar structural damage, pulmonary oedema, inflammation, and fibrosis [3], which are the histological features of bronchopulmonary dysplasia (BPD). Thus, early treatment with NCPAP [1] and surfactant [4] which decreases the need for MV may be an effective strategy for reducing the incidence of BPD in preterm infants with RDS [5-7].

Recently, some randomised controlled studies have investigated the issue of early respiratory management of preterm infants [8-11], and in particular the role of NCPAP and prophylaxis with surfactant in preventing MV and decreasing mortality and/or BPD. A meta-analysis based on some of these studies [10,11] concluded that early treatment with NCPAP combined with the "rescue" surfactant administration to infants requiring intubation has similar effects to prophylactic surfactant treatment on infants’ outcomes, supporting the use of surfactant as the early selective treatment [12].

On the other hand, the best criteria for surfactant treatment in the perinatal period are unknown, and the investigation on the issue of early versus late surfactant treatment is ongoing. Verder *et al*. demonstrated in 1999 that early treatment with surfactant (median age 5.5 hours of life), administered during a short period of intubation to infants with an a/APO_2_ of 0.35 - 0.22 (FiO_2_ of 0.37- 0.55), in comparison with late treatment (median age 9.9 hours of life) in infants with an a/APO2 of 0.21 – 0.15 (FiO_2_ of 0.57– 0.77), decreased the need for MV and/or mortality [13]. The most recent guidelines recommend that babies with RDS should be given rescue surfactant early in the course of the disease [14].

The hypothesis of this study is that a strategy of early treatment with surfactant, in addition to being clinically more effective, is also economically advantageous in comparison to a strategy of late treatment. To test this hypothesis we performed an economic evaluation of the two strategies using clinical data from the Verder *et al*. study [13] and costs calculated in the Neonatal Intensive Care Unit of an Italian hospital.

## Materials and methods

### The reference study

The cost-effectiveness analysis was carried out analysing the results of the randomised controlled Verder *et al*. study [13]. In this study it was shown that treatment of infants of <30 weeks gestational age using the INSURE (INtubation-SURfactant-Extubation) procedure with surfactant (Curosurf® 200 mg/kg, Chiesi Farmaceutici SpA, Parma, Italy), performed *early* (5.2 hours of life), at an a/APO_2_ value of 0.35-0.22, equal to FiO_2_ of 0.37-0.55, significantly reduces [21% vs. 63%, p <0.005] the need for MV or mortality during the first 7 days of life compared to *late* treatment (9.9 hours old), performed at an a/APO2 value of 0.21-0.15, equal to an FiO_2_ of 0.57-0.77 [13]. Table 1 shows the clinical features of patients assigned to the two treatment groups; the patients did not show significant differences at randomisation. In Table 2 the primary and secondary *endpoints* are shown and other variables that showed significant differences between the two groups. Lastly, Table 3 shows the duration of oxygen therapy, of NCPAP and of MV in the two treatment groups.

**Table 1:** Clinical characteristics of patients at randomisation

	**Early treatment**	**Late treatment**	***P***
	**(n = 33)**	**(n = 27)**	
Weight at birth (grams)	950 (665–1600)	935 (618–1555)	*0.84*
Gestational age (weeks)	27 (25–29)	28 (25–29)	*0.54*
Age (hours)	4.1 (0.3-40.1)	4.5 (1.7-41.3)	*0.55*
a/APO_2_	0.28 (0.22-0.38)	0.28 (0.08-0.77)	*0.69*
TcPCO_2_ (mm Hg)	50 (34–103)	47 (30–74)	*0.69*

The values are expressed as median and (range).

**Table 2:** **Endpoint of the clinical study in *early *** **and *late *** **treatment groups**

	***Early *** **treatment**	***Late *** **treatment**	***P***
	**(n = 33)**	**(n = 27)**	
MV and/or death < 7 days, number (%)	7 (21)	17 (63)	*0.0013*
MV and/or death prior to discharge, number (%)	9 (27)	19 (70)	*0.004*
MV prior to discharge, number (%)	8 (25)	17 (68)	*0.005*
a/APO_2_ after 6 hours, mean ± SD	0.48 ± 0.18	0.36 ± 0.18	*0.02*
*Patent ductus arteriosus*, number (%)	10 (30)	16 (59)	*0.02*

**Table 3:** **Duration of oxygen therapy, NCPAP and MV in " *early *** **" and " *late *** **" treatment groups**

	***Early *** **treatment**	***Late *** **treatment**	***P***
	**(n = 33)**	**(n = 27)**	
Oxygen therapy (days)	6.5 (0.3-69)	18.5 (0.5-64)	*0.54*
NCPAP (days)	38.5 (0.8-64)	39.0 (12–155)	*0.76*
MV (days)	2.5 (1.2-5.5)	2.1 (0.3-13.9)	*0.47*

The values are expressed as median and (range).

### Methods

We conducted the (incremental) cost-effectiveness analysis of *early* strategy compared to *late* strategy to assess clinical efficacy and costs of the principal healthcare resources employed during hospitalisation for respiratory care, with the purpose of analyzing them by the hospital perspective of direct medical costs. In particular, the costs of the surfactant, the depreciation of the ventilators used and all the consumable materials needed for respiratory assistance, as well as the medical and nursing staff costs, were taken account of. The latter costs were calculated taking account of the average time needed to perform the various relief operations (e.g., endotracheal aspiration).

Other direct medical costs related to treatment were not considered, as assumed to be similar for the two therapeutic approaches in consideration (*early* vs. *late*) [15]. All costs were estimated in Euro (€) relating to the year 2013. The clinical efficacy and the costs estimated both occurred during the period of hospital admission which, based on what was reported in the clinical trial of reference [13], concluded in a period of time of less than one year. For this reason costs were estimated at the current value without applying any discount rate [15,16].

### Consumption and costing of health resources

According to what was shown in the Verder *et al*. study [13], the surfactant (Curosurf®, Chiesi Farmaceutici SpA, Parma, Italy) was administered at a dose of 200 mg/kg for the first time. Given an average weight at birth of 950 grams for the *early* group and 935 grams for the *late* group, it can be calculated that the first dose of surfactant was on average equal to 190 grams (33/33 patients,100%) and 187 grams (18/27 patients, 67%) in the two groups respectively. The second dose was instead equal to 100 mg/kg and administered in 9% (3/33) of *early* group infants and 11% (3/27) of *late* group infants. As the weight of the infants was not known at the time of the second dose, an average of 95 mg dose of surfactant for the *early* group and 93.5 mg for the *late* group was estimated, based on weight at birth [13]. The acquisition cost of the surfactant was calculated based on the hospital final purchase price updated to July 2013 and equal to Euro 2.31 per milligram of product.

The daily cost of treatment with NCPAP or MV was calculated with reference to what was highlighted by a previous study [17], thanks to which it was possible to estimate the consumable materials and duration of doctor/nurse assistance needed to perform NCPAP or MV, distinguishing between the first day, during which the circuit is installed (for NCPAP and for MV), and subsequent days, when maintenance and monitoring of ventilation systems is performed instead. Tables 4 and 5 respectively show the costs of NCPAP and of MV, differentiating between the first and subsequent days and taking account of the type and quantities of consumable materials used, as well as the unit costs and the time employed by professionals (doctor and nurse).

**Table 4:** Unit cost of materials and of care staff for treatment with NCPAP (%)

	**Unit costs**	**First day**	**First day cost**	**Subsequent days**	**Subsequent days cost**
***Consumable materials***		***(n)***		***(n)***	
Ventilator*	€ 1.37	1	€ 1.37	1	€ 1.37
Patient ventilator circuit	€ 95.83	1	€ 95.83	0.17	€ 15.97
Canopy for gas humidification	€ 12.71	1	€ 12.71	0.17	€ 2.12
Hood for generator	€ 20.32	1	€ 20.32	-	-
Nasal mask for generator	€ 17.90	1	€ 17.90	-	-
500 ml H_2_O humidifier	€ 0.85	1	€ 0.85	1	€ 0.85
Gold/nasal disposable aspiration tube	€ 0.32	8	€ 2.56	8	€ 2.56
Anti-decubitus nasal hydrocolloid plaques	€ 0.60	1	€ 0.60	-	-
***Consumable materials total***			***€ 152.13(66)***		***€ 22.87(21)***
***Staff***		***(minutes)***		***(minutes)***	
Mounting of ventilator and circuit	€ 0.43	15	€ 6.50	-	-
Positioning (5 minutes x 8 times)	€ 0.43	40	€ 17.33	40	€ 17.33
Gold pharyngeal aspiration (8 asp.)	€ 0.43	15	€ 6.50	15	€ 6.50
Direct care and equipment monitoring	€ 0.43	100	€ 43.33	100	€ 43.33
Radiography (1 die)	€ 0.43	10	€ 4.33	10	€ 4.33
Substitution of respiratory circuit	€ 0.43	-	-	35	€ 15.08
***Staff care total***			***€ 78.00(34)***		***€ 86.58(79)***
**Total**			**€ 230.13**		**€ 109.45**

*Infant Flow® SiPAP System, San Diego, CA, USA.

**Table 5:** Unit cost of materials and of care staff for treatment with MV (%)

	**Unit costs**	**First day**	**First day cost**	**Subsequent days**	**Subsequent days cost**
***Consumable materials***		***(n)***		***(n)***	
Ventilator*	€ 9.59	1	€ 9.59	1	€ 9.59
Leoni Plus patient ventilator circuit	€ 104.54	1	€ 104.54	0.17	€ 17.42
Endotracheal tube	€ 1.06	1	€ 1.06	0.5	€ 0.53
500 ml H_2_O humidifier	€ 0.85	1	€ 0.85	1	€ 0.85
Closed circuit for E.T. aspiration	€ 13.18	1	€ 13.18	0.5	€ 6.59
Gold/nasal disposable aspiration tube	€ 0.32	8	€ 2.56	8	€ 2.56
Disposable syringe (for sedation)	€ 0.21	1	€ 0.21	1	€ 0.21
Set for i.v. infusion (for sedation)	€ 0.20	1	€ 0.20	1	€ 0.20
***Consumable materials total***			***€ 132.19(57)***		***€ 37.95(28)***
***Staff***		***(minutes)***		***(minutes)***	
Mounting of ventilator and circuit	€ 0.43	15	€ 6.50	-	-
Positioning (5 minutes x 8 times)	€ 0.43	40	€ 17.33	40	€ 17.33
E.T. aspiration (3 – 8 asp.)	€ 0.43	20	€ 8.67	20	€ 8.67
Gold pharyngeal aspiration (8 asp.)	€ 0.43	15	€ 6.50	15	€ 6.50
Direct care and equipment monitoring	€ 0.43	60	€ 26.00	60	€ 26.00
Continuous intravenous sedation	€ 0.43	15	€ 6.50	15	€ 6.50
Radiography (2 die)	€ 0.43	20	€ 8.67	20	€ 8.67
Intubation assistance**	€ 1.00/0.43	20	€ 20.00	20	€ 8.76
Substitution of respiratory circuit	€ 0.43	-	-	35	€ 15.08
***Staff care total***			***€ 100.17(43)***		***€ 97.41(72)***
**Total**			**€ 232.35**		**€ 135.36**

*Leoni Plus®, Heinen + Lowenstein, Bad Ems, Germany.

**Doctor assistance is expected on the first day (costed at an hourly cost of € 60.00), while for subsequent days the assistance of nursing staff only is assumed (costed at an hourly cost of € 26.00).

The daily depreciation cost of the ventilation system, amounting to Euro 1.37 for NCPAP and Euro 9.59 for MV, was calculated by reference to the hospital purchase price (NCPAP: Infant Flow® SiPAP System, San Diego, CA, USA, Euro 5,000; MV: Leoni Plus®, Heinen + Lowenstein, Bad Ems, Germany, Euro 35,000) divided by the duration of the asset depreciation process (10 years, equivalent to 3,650 days). All other materials used for the provision of NCPAP or MV were costed based on their market prices. For the costing of staff, only the presence of a professional nurse was considered in treatment with NCPAP, while for MV, the professional figure of a doctor, when required, was also considered. The hourly cost for professionals, as indicated by the Italian national labour collective agreement, was calculated at Euro 26.00 for the nurse (cost per minute Euro 0.43) and Euro 60.00 for the doctor (cost per minute Euro 1.00).

The cost of NCPAP was calculated by multiplying the number of days of service provision (*early* group: 38.5 days; *late* group: 39 days) by the unit cost (distinguishing between the first and subsequent days) multiplied by the frequency of the event itself, which in this specific case was of 100% of patients in both groups. The same method of calculation was used to determine the average cost of MV per patient, which showed a different frequency (*early* group: 25%; *late* group: 68%) and duration (*early* group: 2.5 days; *late* group: 2.1 days) between the two groups. To check the effects of a change in value of the main variables in calculation of *early* and *late* strategy costs, we simulated the effects of a change of plus or minus 15% and 30% in the costs of NCPAP and of MV. In addition, we simulated the effects of an increase of 15%, 30% or 50% in the number of patients who required MV and of the MV duration in the *early* group.

## Results

The cost of consumable material was greater for NCPAP (Euro 152.13) than for MV (Euro 132.19) for the first day of treatment, while for subsequent days it was greater for MV (Euro 22.87 vs. Euro 37.95). The cost of staff was greater for MV compared to NCPAP both for the first day (Euro 100.17 vs. Euro 78.00) and for subsequent days (Euro 97.41 vs. Euro 86.58). The overall cost of the first day of treatment was equal to Euro 230.13 for NCPAP and Euro 232.35 for MV, while the cost for each subsequent day was Euro 109.45 for NCPAP and Euro 135.36 for MV (Tables 4 and 5).

The cost of treatment with surfactant was Euro 438.56 in the *early* group and Euro 287.76 in the *late* group, while the cost of MV was Euro 108.85 in the *early* group and Euro 259.25 in the *late* group. The average cost for patients treated with the *early* strategy was moderately lower than for patients treated with the *late* strategy (Euro 4,901.70 vs. Euro 4,960.07), a difference of Euro 58.37 (Table 6).

**Table 6:** **Comparison of cost-effectiveness (€) in " *early *** **" and " *late *** **" treatment groups**

	***Early *** **treatment**	***Late *** **treatment**	**Difference**
**Primary Endpoint**			
MV and/or death < 7 days, (%)	21	63	42
**Average cost per patient treated**			
- surfactant (1st^.^ dose)	438.56	287.76	150.80
- surfactant (2nd^.^ dose)	19.93	23.98	4.05
- NCPAP	4,334.36	4,389.08	54.72
- MV	108.85	259.25	150.40
Total	4,901.70	4,960.07	58.37

The simulation of changes in the cost of NCPAP and MV were associated always with a lower cost for *early* strategy compared to *late* strategy. The simulation of an increase in the number of patients who required MV and of MV duration in the group was associated always with a lower *early* strategy cost compared to *late* strategy cost (Table 7).

**Table 7:** One-way (univariate) sensitivity analysis

**Parameter**	**"Early" treatment**	**"Late" treatment**	**Difference (€)**	**C/E analysis result**
**Base case**				**Dominant**
MV and/or death < 7 days, (%)*	21	63	-42	
Average cost per treated patient (€)	4,901.70	4,960.07	- 58.37	
**Variation in Mechanical Ventilation cost**				
- plus 15% compared to base case (€)	4,918.03	4,998.95	- 80.93	Dominant
- minus 15% compared to base case (€)	4,885.37	4,921.18	- 35.81	Dominant
- plus 30% compared to base case (€)	4,934.35	5,037.84	-103.49	Dominant
- minus 30% compared to base case (€)	4,869.05	4,882.29	- 13.25	Dominant
**Variation in NCPAP cost**				
- plus 15% compared to base case (€)	5,551.85	5,618.43	- 133.57	Dominant
- minus 15% compared to base case (€)	4,251.55	4,301.70	- 50.16	Dominant
- plus 30% compared to base case (€)	6,202.01	6,276.79	- 74.78	Dominant
- minus 30% compared to base case (€)	3,601.39	3,643.34	- 41.95	Dominant
**% ET patients with MV**				
- plus 15% compared to base case (€)	4,918.03	4,960.07	- 42.04	Dominant
- plus 30% compared to base case (€)	4,934.35	4,960.07	- 25.71	Dominant
- plus 50% compared to base case (€)	4,956.12	4,960.07	- 3.94	Dominant
**ET patient days in MV**				
- plus 15% compared to base case (€)	4,914.39	4,960.07	- 45.68	Dominant
- plus 30% compared to base case (€)	4,927.08	4,960.07	- 32.99	Dominant
- plus 50% compared to base case (€)	4,944.00	4,960.07	- 16.07	Dominant

*For calculation of the cost-effectiveness ratio the reciprocal of the estimated given efficacy by the Verder *et al.* study was considered [12], i.e., the percentage of patients that do not undergo MV and/or death within seven days of birth equal to 79% for the ET group and 37% for the LT group was considered.

Because *early* treatment was the dominant therapeutic option since more effective and less costly than *late* treatment (Table 7), it was not necessary to calculate the incremental cost-effectiveness ratio between the two alternatives considered here.

## Discussion

The objective of this study was the analysis of the costs (hospital’s perspective) of two strategies of surfactant administration, *early* vs. *late,* proposed in the Verder *et al*. study [13], using the experience of an Italian NICU for calculation of the costs. In particular, only the costs associated with treatment of respiratory failure in preterm infants with RDS were considered, i.e., those costs related to administration of surfactant and to the frequency and duration of NCPAP and/or MV treatment, as these represent the most important difference in care that emerged between the two treatment alternatives. We have shown that the costs of *early* treatment (Euro 4,901.70) are substantially similar (slightly lower) than those of *late* treatment (Euro 4,960.07). For this reason, considering that the Verder study [13] demonstrated greater clinical efficacy of *early* treatment, reducing the frequency of MV and/or mortality, this result further confirms the benefits of such a strategy and supports its application.

Analysing the costs in detail, it may be observed how in the *early* group the cost of treatment with surfactant is greater (Euro 458.49 vs. Euro 311.74). However, this is offset by the higher cost of treatment with MV in the *late* group (Euro 259.25 vs. Euro 108.85), because in this group the frequency of MV (68% vs. 25%) was higher. In both groups the main cost is represented by NCPAP, equal to 88% of the total cost, an expected result considering that all the enrolled patients received this treatment, the duration of which was similar in the two groups (39 vs. 38.5 days).

Analysing the NCPAP and MV costs, it is interesting to note that the cost of material for the first day of treatment is greater for NCPAP than for MV, while for subsequent days the opposite occurs. With regard to the cost of staff, this is greater in MV than in NCPAP, both in the first and subsequent days. Of importance also is how the cost of staff decreases after the first day of MV while increasing after the first day of NCPAP. In summary, the cost of material for beginning NCPAP is greater than for beginning MV, while the cost of material for continuing NCPAP is less; the cost of staff for beginning and continuing MV is greater than for beginning and continuing NCPAP.

To evaluate and confirm these results it would have been very useful to have been able to compare them with other similar studies, but unfortunately a similar cost analysis of respiratory support with surfactant, NCPAP and MV in preterm infants with RDS has never been carried out before.

To work around this limitation and therefore attempt to confirm our results, we carried out a simulation of different scenarios in which the cost of NCPAP and of MV was increased or decreased by 15% and by 30% and the frequency and duration of NCPAP and of MV increased by 15%, 30% and 50%. Even in these cases, the *early* strategy was found to be the dominant alternative (Table 7).

A limitation of this economic analysis may be in having used the clinical results of a study carried out in Denmark [13], which may not be transferable to the Italian population. This choice was justified by a shortage in the literature of randomised controlled trials that compare the efficacy of *early* vs. *late* surfactant treatment. In fact, of the six published studies two were performed using synthetic surfactant [18,19] and two using bovine surfactant [20,21], while in Italy only porcine surfactant is commercially available. Furthermore, another study was conducted in a cohort of patients who were all in MV with high frequency oscillatory ventilation (HFOV) [22], while none of these studies [18-22] included the use of the INSURE procedure, widely used in Italy [23]. Therefore, the Verder study [13], both for the type of surfactant used as well as for the management of respiratory failure, is the one which can be considered most similar to the management of preterm infant RDS in our country, even though the thresholds of *early* (FiO_2_ of 0.37-0.55) and *late* (FiO_2_ of 0.57-0.77) treatment are far greater than those currently in use in Italy.

## Conclusions

Despite its simplifications, the cost-effectiveness analysis performed in this study demonstrates how *early* treatment with surfactant of preterm infants with RDS is not only more effective clinically, but is also economically cheaper than *late* treatment. In fact, the greater initial costs of *early* treatment with surfactant are compensated by subsequent lower costs required for MV. These results must find confirmation, however, in future randomised and controlled studies that can perform a comparison in a prospective mode.

## Competing interests

MC is an employee of Chiesi Farmaceutici S.p.A. RR, CD and LF declare that they have no competing interests.

## Authors’ contributions

All the Authors contributed to study conception, data acquisition and analysis, drafting of the manuscript and approval of the final draft.
